# Expression of the herpes simplex virus type 1 latency-associated transcripts does not influence latency establishment of virus mutants deficient for neuronal replication

**DOI:** 10.1099/vir.0.056176-0

**Published:** 2013-11

**Authors:** M. P. Nicoll, S. Efstathiou

**Affiliations:** Division of Virology, Department of Pathology, University of Cambridge, Tennis Court Road, Cambridge CB2 1QP, UK

## Abstract

Herpes simplex virus type 1 establishes latency within neurons of the trigeminal ganglion. During latency, viral gene expression is largely restricted to the latency-associated transcripts (LATs), which, whilst not essential for any aspect of latency, function to suppress lytic gene expression and enhance the survival of virus-infected neurons. The latent cell population comprises primary-order neurons infected directly from peripheral tissues and cells infected following further virus spread within the ganglion. In order to assess the role of LAT expression on latency establishment within first-order neurons, we infected ROSA26R reporter mice with Cre recombinase-expressing recombinant viruses harbouring deletion of the thymidine kinase lytic gene and/or the core LAT promoter. We found that LAT expression did not impact on latency establishment in viruses unable to replicate in neurons, and under these conditions, it was not required for the survival of neurons between 3 and 31 days post-infection.

Following primary mucosal infection, herpes simplex virus type 1 (HSV-1) gains access to sensory neurons of the trigeminal ganglion (TG) and establishes a latent infection. During latency, transcription of the virus genome is highly restricted and is largely limited to the latency-associated transcript (LAT), a non-protein-coding 8.3 kb mRNA encoded in repeat sequences flanking the U_L_ region of the HSV-1 genome (Rock *et al.*, 1987; Stevens *et al.*, 1987). This primary transcript is processed into 1.5 and 2 kb stable introns (termed major LATs) (Zabolotny *et al.*, 1997) and an unstable 6.3 kb (minor LAT) exon, the latter of which is further processed into numerous microRNAs (Jurak *et al.*, 2010; Umbach *et al.*, 2008). Whilst studies using small-animal models have demonstrated that LATs are not essential for latency (Javier *et al.*, 1988; Steiner *et al.*, 1989), these transcripts have been shown to influence the efficiency by which HSV-1 establishes (Nicoll *et al.*, 2012; Perng *et al.*, 2000b; Thompson & Sawtell, 1997) and/or reactivates from (Hill *et al.*, 1990; Leib *et al.*, 1989; Perng *et al.*, 1994) latent infections. Furthermore, recent analyses have also provided evidence that the latent cell reservoir is less stably maintained (Nicoll *et al.*, 2012) and loses long-term reactivation competence (Thompson & Sawtell, 2011) in the absence of LAT expression. The cellular and molecular basis of such LAT functions is less forthcoming, but is believed to centre on potential roles for the LAT in promoting cell survival (Branco & Fraser, 2005; Perng *et al.*, 2000a; Thompson & Sawtell, 2001) and inhibiting immediate-early (IE) viral lytic gene expression by post-transcriptional and/or epigenetic mechanisms (Cliffe *et al.*, 2009; Umbach *et al.*, 2008; Wang *et al.*, 2005). Indeed, in the absence of LAT expression, a greater frequency of TG cells expressing HSV-1 IE and early (E) gene transcripts can be observed by *in situ* hybridization during acute infection in mice (Garber *et al.*, 1997), and increased lytic gene expression can also be detected during latency (Chen *et al.*, 1997). It is not clear whether such an increase in virus lytic gene expression results in greater spread of the virus throughout neuronal tissue during acute infection, or indeed whether such a spread may result in an increased frequency of latently infected neurons. It has been found previously that HSV-1 LAT-negative mutants establish latency in fewer neurons relative to LAT-positive virus in the mouse TG (Thompson & Sawtell, 1997, 2001), a phenotype we have also observed following infection of the mouse whisker pad at high virus doses (Nicoll *et al.*, 2012). In contrast to these data, we have also reported that HSV-1 recombinants deficient for LAT expression (due to deletion of the core latency-associated promoter, or LAP) established latency in ~33 % more neurons per TG relative to revertant virus (following infection of mouse whisker pads with 10^6^ p.f.u.; Nicoll *et al.*, 2012), suggesting that LAT expression may repress the replication and spread of HSV-1 within the TG. Despite this, we have been unable to observe LAT-dependent differences in virus replication within the TGs by standard assays of virus infectivity within BALB/c or ROSA26R mouse strains (Nicoll *et al.*, 2012). The efficiency of latency establishment is influenced by numerous factors including: (i) the extent of peripheral replication; (ii) the number of primary-order neurons initially infected; (iii) the fate of infected neurons; and (iv) the extent of spread to neuronal cells within the ganglion that do not directly innervate the site of primary infection. Given the multitude of factors that can influence latent loads, we re-examined the establishment phenotype of LAT-positive and LAT-negative viruses with recombinants incapable of lytic replication within sensory neurons.

To examine the efficiency of latency establishment of LAT-positive and LAT-negative mutants in first-order neurons, we introduced a previously characterized deletion of the HSV-1 thymidine kinase (TK) gene (Fig. 1a) from HSV-1 SC16 recombinant TKDM21 (Efstathiou *et al.*, 1989) into Cre reporter viruses HSV CMVCre [CMVCre carries the WT *cre* recombinase gene under the control of the major IE promoter and enhancer of human cytomegalovirus (CMV)], HSV CMVCreΔLAT–GFP (in which the 203 bp core LAP is replaced with a GFP expression cassette in the opposite orientation to the LAT transcript) and HSV CMVCreREV (a revertant virus of HSV CMVCreΔLAT–GFP) (Nicoll *et al.*, 2012). HSV-1 mutants deleted for TK activity have been shown previously to replicate within epithelial tissues but are severely diminished or incapable of replication within sensory ganglia (Coen *et al.*, 1989; Efstathiou *et al.*, 1989; Thompson & Sawtell, 2000). Detection of lytic gene antigens during acute infection with TK-negative viruses suggests that this results from a deficit for viral DNA replication, as IE proteins, but not true late gene proteins, can be detected within mouse TGs (Sawtell *et al.*, 2006). Thus, by inhibiting viral DNA replication in the sensory neurons of the TG, but not in cells at the periphery, the role of virus replication with regard to the frequency of latency establishment was assessed for both LAT-positive and LAT-negative viruses.

**Fig. 1.**
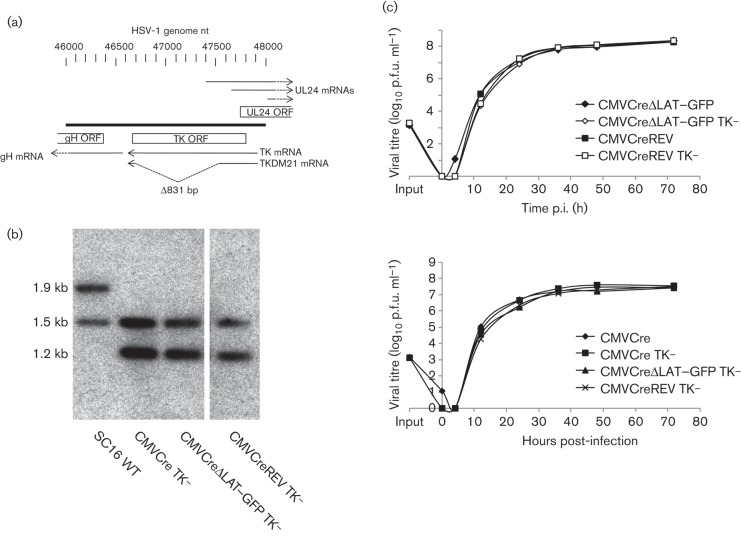
(a) Structure of the HSV-1 U_L_22 (gH), U_L_23 (TK) and U_L_24 loci. Within TKDM21, an 831 bp deletion in the TK gene is present between nt 46695 and 47526 of the TK ORF. Number scale represents HSV-1 nucleotides (GenBank accession no. NC_001806) (McGeoch *et al.*, 1988). (b) Genomic structures as analysed by Southern blot hybridization. Restriction digest with *Eco*RV demonstrated the presence of the TKDM21 deletion within HSV CMVCre TK^−^, HSV CMVCreΔLAT–GFP TK^−^ and HSV CMVCreREV TK^−^ and lack of aberrant rearrangements within in the U_L_22–U_L_25 loci. *Eco*RV digestion of WTSC16 also produced a 104 bp restriction fragment (not shown). (c) Independent low m.o.i. (0.01) *in vitro* growth curves of WT TK and TK-deleted HSV CMVCre, HSV CMVCreΔLAT–GFP and HSV CMVCreREV, conducted on BHK cells, at the indicated time p.i.

To generate the recombinant viruses HSV CMVCre TK^−^, HSV CMVCreΔLAT–GFP TK^−^ and HSVCMVCreREV TK^−^, infected-cell DNA prepared from HSV CMVCre, HSV CMVCreΔLAT–GFP and HSV CMVCreREV, respectively, was co-transfected into baby hamster kidney (BHK) cells with *Sca*I-linearized pUC19-TKDM21. To construct pUC19-TKDM21, PCR primers (forward, 5′-AAAAATAAGCTTAGCAGGTAGGTCTTCGG-3′; reverse, 5′-AAAAATAAGCTTGAGCTTCAGGGAGTGGC-3′) directing amplification between WT HSV-1 nt 46055 and 48113 (GenBank accession no. NC_001806; McGeoch *et al.*, 1988) were utilized to clone the mutant TK-coding sequence from HSV-1 TK-negative recombinant TKDM21 (Efstathiou *et al.*, 1989) into pUC19. Recombinant progeny containing the TKDM21 sequence were enriched twice by infection at a low m.o.i. of 0.01 of BHK monolayers in Dulbecco’s modified Eagle’s medium containing 10 % FCS and supplemented with 5 µg acyclovir ml^−1^. Recombinants were then purified by three rounds of limiting dilution infection in the presence of acyclovir and selected by PCR for the presence of an 831 bp deletion in the TK-coding sequence (data not shown). The presence of a TK deletion in all three recombinants was confirmed by Southern blot hybridization (Fig. 1b). All recombinants were observed by PCR to have retained the corresponding LAP genotype of their parental viruses (Nicoll *et al.*, 2012) (data not shown). The *in vitro* replication kinetics of HSV CMVCre TK^−^, HSV CMVCreΔLAT–GFP TK^−^ and HSV CMVCreREV TK^−^ were similar, as well as being indistinguishable from each parental virus (Fig. 1c) following infection of BHK cell monolayers at a low m.o.i. of 0.01.

The TKDM21 mutation was demonstrated previously to render HSV-1 incapable of replication in cervical dorsal root ganglia, whilst retaining the ability to replicate within inoculated ear tissue, albeit at reduced titres throughout the duration of acute infection (Efstathiou *et al.*, 1989). To ascertain whether an analogous phenotype would be observed following whisker pad infection, BALB/c mice were inoculated under isoflurane anaesthesia with HSV CMVCre, HSV CMVCre TK^−^, HSV CMVCreΔLAT–GFP, HSV CMVCreΔLAT–GFP TK^−^, HSV CMVCreREV or HSV CMVCreREV TK^−^ by light scarification with 10^6^ p.f.u. per whisker pad, as described previously (Nicoll *et al.*, 2012). Whisker pads and TGs were dissected at 4 days post-infection (p.i.), homogenized and assayed for infectious virus on BHK cell monolayers. All TK-deletion viruses achieved similar titres within whisker pad tissues, but were ~1 log lower than TK-positive viruses (Fig. 2a). Together, these data demonstrate that in the absence of TK activity each recombinant was competent for replication in whisker pad tissues, but displayed attenuation that was consistent across all TK-negative viruses. In contrast, no infectious virus was recovered from the TGs of mice infected with HSV CMVCre TK^−^, HSV CMVCreΔLAT–GFP TK^−^ and HSV CMVCreREV TK^−^, whilst >1000 p.f.u. could be recovered from all three parental TK-positive viruses (Fig. 2a). To confirm these observations, the experiment was repeated in ROSA26R mice with HSV CMVCre, HSV CMVCre TK^−^, HSV CMVCreΔLAT–GFP TK^−^ and HSV CMVCreREV TK^−^ (five mice per virus). As with the BALB/c mice, TK-negative virus infectivity could not be detected within the TGs, whilst a mean (±sem) of 4.2×10^3^±7.5×10^2^ p.f.u. of TK-positive HSV CMVCre was recovered at 4 days p.i. (Fig. 2b). Again, all TK-negative virus titres were comparable within whisker pad tissues (Fig. 2b), confirming that lytic replication was restricted to whisker pad tissue, in agreement with a previous study in which infectious virus was only infrequently detected in the TGs (and at <8 p.f.u. per positive ganglion at 4 days p.i.) following snout inoculation with a TK-null mutant (Thompson & Sawtell, 2000).

**Fig. 2.**
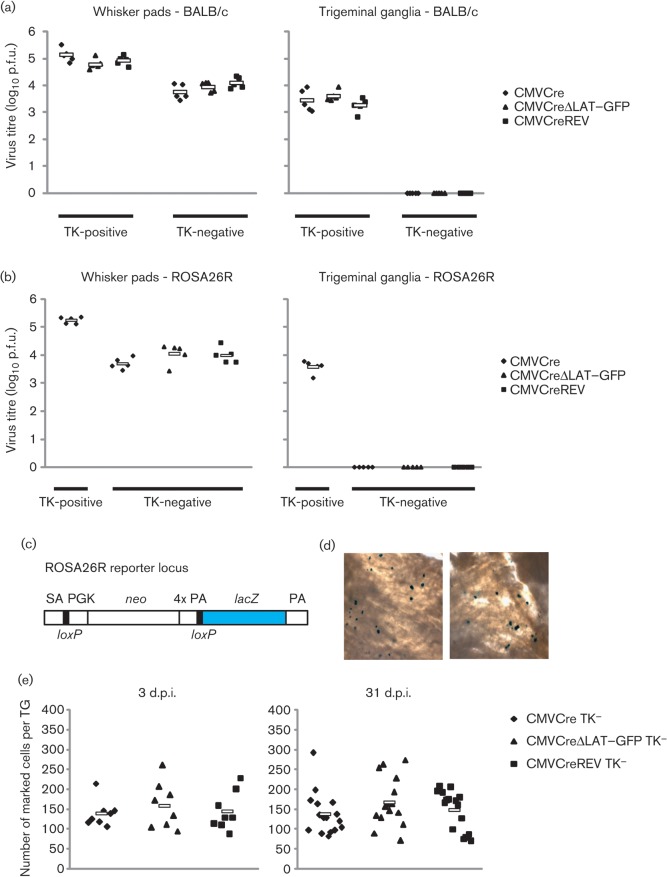
(a) Titres of TK-positive (HSV CMVCre, HSV CMVCreΔLAT–GFP and HSV CMVCreREV) and TK-negative (HSV CMVCre TK^−^, HSV CMVCreΔLAT–GFP TK^−^ and HSV CMVCreREV TK^−^) recombinants within BALB/c whisker pad and TG pairs at 4 days p.i. (b) Titres of TK-positive (HSV CMVCre) and TK-negative (HSV CMVCre TK^−^, HSV CMVCreΔLAT–GFP TK^−^ and HSV CMVCreREV TK^−^) recombinants within ROSA26R whisker pad and TG pairs at 4 days p.i. In (a) and (b), each symbol represents the titre from an individual mouse and the floating bar displays the mean titre from each virus-infected group. (c) Structure of the ROSA26 locus in R26R reporter mice. The transgene contains a splice acceptor sequence upstream of a neomycin phosphotransferase gene (*neo*) and four poly(A) (PA) signal sequences (together flanked by *loxP* sites) and a downstream *lacZ* gene. Following Cre-driven recombination of the *loxP*-flanked cassette, *lacZ* is constitutively expressed by the ROSA26 promoter. (d) Photomicrographs demonstrating the presence of marked cells following dissection, fixation and incubation of TGs with X-Gal. (e) Infected-cell marking from ROSA26R mice at 3 and 31 days following infection with HSV CMVCre TK^−^, HSV CMVCreΔLAT–GFP TK^−^ and HSV CMVCreREV TK^−^. Each symbol represents the number of marked cells in an individual TG and the floating bar displays the mean number from each virus-infected group.

Having confirmed that all three TK-deleted recombinants did not replicate to detectable levels within TGs, we next assessed infected-cell marking during acute and latent infection using the previously characterized ROSA26R reporter mouse model of HSV-1 infection (Proença *et al.*, 2008; Wakim *et al.*, 2008). In this model, the human CMV major IE promoter-controlled expression of Cre recombinase from the infecting virus leads to the permanent genetic marking of latently infected neurons within the reporter mouse. This is due to Cre-mediated excision of a *loxP*-flanked neomycin resistance gene cassette situated between the constitutive ROSA26 promoter and a downstream *lacZ* reporter gene within the transgenic mouse (Fig. 2c). As a result, *lacZ* is expressed constitutively from the mouse genome, allowing visualization and enumeration of latently infected cells following incubation with the substrate X-Gal (Fig. 2d). Analyses of individually marked neurons by PCR for viral DNA have shown that all marked cells are virus DNA positive, supporting the view that the frequency of marked neurons accurately reflects latent loading of sensory ganglia (unpublished observations). Thirty-six ROSA26R mice were inoculated under isoflurane anaesthesia with 10^6^ p.f.u. per whisker pad of HSV CMVCre TK^−^, HSV CMVCreΔLAT–GFP TK^−^ or HSV CMVCreREV TK^−^ in groups of twelve animals. At 3 days p.i., four mice from each group were killed and the TG tissues dissected. Both TGs from each mouse were pooled, fixed in ice-cold 4 % paraformaldehyde for 90 min, rinsed with PBS and incubated overnight at 37 °C in X-Gal, as described previously (Lachmann & Efstathiou, 1997; Proença *et al.*, 2011). The TGs were then photomicrographed and marked cells were counted in a blinded manner to avoid experimental bias. At 3 days p.i., the mean number of marked cells was comparable for each virus, with 131.5 (range 105–214), 157.9 (range 94–260) and 143.8 (range 86–228) marked cells per TG for HSV CMVCre TK^−^, HSV CMVCreΔLAT–GFP TK^−^ and HSV CMVCreREV TK^−^, respectively (Fig. 2e). No significant divergence was observed among the three marked cell populations (*P* = 0.83; Kruskal–Wallis test). The remaining eight mice per group were killed at 31 days p.i. (a time point consistent with latency) and the TGs were dissected and treated as above. Once again, the marked cell populations were comparable among all three viruses; 137.2 (range 88–292), 166.9 (range 89–262) and 146.6 (range 99–206) marked cells per TG for HSV CMVCre TK^−^, HSV CMVCreΔLAT–GFP TK^−^ and HSV CMVCreREV TK^−^, respectively (Fig. 2e), and no significant difference was observed between the three marked cell populations (*P* = 0.41; Kruskal–Wallis test). Marked cell populations were highly comparable in three further independent experiments (Table 1). The mean of each marked cell population was reduced by fourfold to fivefold compared with analogous experiments with each TK-positive parental virus (Nicoll *et al.*, 2012). Such differences are similar to those reported by Thompson & Sawtell (2000), in which the percentage of TG neurons latently infected by an HSV-1 TK mutant were approximately sixfold less than rescued virus following snout inoculation. Furthermore, in comparison with our previous data in which TK-positive LAT-negative viruses established latency in ~33 % more neurons relative to LAT-revertant virus (at an infectious dose of 10^6^ p.f.u. per whisker pad; Nicoll *et al.*, 2012), the absence of an establishment phenotype in analogous experiments with TK-negative mutants supports the view that the enhanced frequency of latency establishment of LAT-negative mutants is dependent on virus replication within neuronal cells. These data thus provide indirect evidence that LAT expression may actively limit the number of neurons in which latency is established. The mechanism for this phenotype is probably the hindrance of lytic expression, virus replication and spread within the TG itself. This hypothesis is supported by a study from Garber and colleagues, in which *in situ* hybridization was utilized to demonstrate increased lytic gene expression in the mouse TGs from LAT-deleted virus, relative to WT HSV-1 (Garber *et al.*, 1997). Whilst Garber and colleagues did not demonstrate a concordant increase in virus titres, it is notable that a decrease in IE gene expression and HSV-1 replication was observed in mouse neuroblastoma cell lines stably expressing the primary LAT (either in its entirety or with the sequence 3′ of the LAT 2 kb intron deleted; Mador *et al.*, 1998). As it has been demonstrated that HSV-1 reactivation efficiency is positively correlated with latent cell reservoir size (Sawtell, 1998), such a restriction by the LAT may seem counterproductive to efficient colonization of the host and future transmission. However, a limitation to virus replication in the peripheral nervous system could serve to prevent uncontrolled spread and lethal central nervous system infection, a strategy compatible with continued long-term virus transmission.

**Table 1.** Marked cell populations during latent infection with TK-negative HSV-1 recombinants in four independent experiments ‘All’ represents an aggregate of the data.

Experiment no. (days p.i.*)	Mean marked cell number per TG (range)			*P*†	*n*‡
CMVCre TK^−^	CMVCreΔLAT–GFP TK^−^	CMVCreREV TK^−^
1 (33)	130.9 (44–181)	127.1 (100–226)	141.4 (82–226)	0.74	8–10
2 (31)	137.2 (82–292)	166.9 (70–273)	146.6 (69–206)	0.41	15–16
3 (29)	–	113.1 (53–202)	77.7 (37–202)	0.07	14
4 (28)	89.6 (37–171)	89.1 (38–151)	–	0.95	16–18
All (28–33)	115.6 (37–292)	123.2 (38–273)	121.2 (37–226)	0.88	40–53

* Days p.i. at which marked cell numbers were assessed.

† *P* value ascertained from non-parametric analyses: Kruskal–Wallis for group-wise comparisons and Mann–Whitney for pairwise comparisons.

‡ Number of individual TGs assessed from each virus group.

Another role attributed to LAT expression is that of promoting infected-cell survival (Branco & Fraser, 2005; Perng *et al.*, 2000a; Thompson & Sawtell, 2001). The LAT could supply such a function either directly on cellular targets or indirectly as a result of limiting virus gene expression and replication. Utilizing TK-deleted viruses, in which virus replication is curtailed in neurons regardless of LAT expression, we observed that marked cell numbers were highly similar for all viruses between 3 and 31 days p.i. (Fig. 2e). These data suggested that infected-cell populations are highly stable and that LAT expression had no measurable effect on cell survival during TK-deleted virus infection.
